# Infamous Attack of the Nashville Journal, upon Dr. Gross and the Jefferson Medical College

**Published:** 1858-09

**Authors:** 


### "INFAMOUS ATTACK OF THE NASHVILLE JOURNAL, UPON DR. GROSS AND THE JEFFERSON MEDICAL COLLEGE."

In our Original Department will be found an article bearing the above title, to which the name of the writer is not attached, in explanation of which we would refer to the article itself.

We think it proper, however, to add (from the unpleasant relations which have existed between the Journal alluded to, and ourselves) that the writer who has assumed the name of "Moxa," has no connection with the Atlanta Medical Journal or College.

So far as we are concerned, in the matter, we have to say, that while we prefer a bona fide name to accompany every article, in the present instance, we have thought proper to consult the preference of the author, whose responsibility is beyond a question.

